# LncRNA BCYRN1-induced autophagy enhances asparaginase resistance in extranodal NK/T-cell lymphoma

**DOI:** 10.7150/thno.46655

**Published:** 2021-01-01

**Authors:** Liang Wang, Jing Yang, He-nan Wang, Rui-ying Fu, Xin-di Liu, Ying-shi Piao, Li-qiang Wei, Jing-wen Wang, Luo Zhang

**Affiliations:** 1 Department of Hematology, Beijing TongRen Hospital, Capital Medical University, Beijing 100730, China; 2 Beijing Advanced Innovation Center for Big Data-Based Precision Medicine, Beihang University & Capital Medical University, Beijing TongRen Hospital, Beijing 100730, China; 3 Department of Pathology, Beijing TongRen Hospital, Capital Medical University, Beijing 100730, China.; 4 Beijing Key Laboratory of Head and Neck Molecular Pathological Diagnosis, Beijing TongRen Hospital, Capital Medical University, Beijing 100730, China.; 5 Department of Otolaryngology Head and Neck Surgery, Beijing TongRen Hospital, Capital Medical University, Beijing 100730, China; 6 Beijing Key Laboratory of Nasal Disease, Beijing Institute of Otolaryngology, Beijing 100730, China

**Keywords:** extranodal NK/T-cell lymphoma, long non-coding RNA, asparaginase, autophagy, resistance

## Abstract

**Background:** Asparaginase (ASP) is the cornerstone drug in the treatment of extranodal NK/T-cell lymphoma (ENKTCL), and the mechanisms of resistance to ASP remain largely unknown. Long non-coding RNAs play important roles in chemotherapy resistance in various cancers. However, the expression of BCYRN1 and its role in ENKTCL still remain unidentified.

**Methods:** Lentivirus-mediated BCYRN1 overexpression and knockdown were performed in SNK-6 cells. Cell autophagy was analyzed by adenovirus expressing GFP-LC3B fusion protein. RNA pull-down and RNA Binding Protein Immunoprecipitation Assay were performed to investigate the relationship between BCYRN1 and p53. Western blot analysis was performed to assess the effect of BCYRN1 on different autophagy pathways. Finally, in vivo xenograft tumor model was constructed to analyze the effect of BCYRN1 on tumor growth and ASP resistance.

**Results:** BCYRN1 was overexpressed in ENKTCL than normal NK cells, and patients with higher expression had significantly inferior progression-free survival (PFS). The IC50 value of ASP was significantly increased in BCYRN1-overexpressed SNK-6 cells and BCYRN1 overexpression could resist the inhibitory effect of ASP on proliferation. ASP could induce concurrent apoptosis and autophagy in ENKTCL, and the latter process was enhanced by overexpression of BCYRN1, mainly through affecting both PI3K/AKT/mTOR and p53/mTOR pathways. BCYRN1 could induce the degradation of p53 via ubiquitination, thus resulting in enhancement of autophagy and ASP resistance, which could be reversed by drug-induced autophagy inhibition. The effect of BCYRN1 on tumor growth and autophagy were confirmed in vivo xenograft model.

**Conclusions:** It was found that BCYRN1 was a valuable prognostic biomarker in ENKTCL. BCYRN1 could promote resistance to ASP by inducing autophagy, which could be reversed by inhibition of autophagy. Our findings highlight the feasibility of combining autophagy inhibition and ASP in the treatment of ENKTCL.

## Introduction

Extranodal NK/T-cell lymphoma, nasal type (ENKTCL) is a highly aggressive, epstein-barr virus (EBV)-associated lymphoma, which is prevalent in East Asia and Middle/South America 1. It has been well recognized that combination of radiotherapy and chemotherapy should be applied in early stage disease, which leads to a 5-year overall survival (OS) rate of about 80% 2. However, patients with advanced disease fare poorly even if stem cell transplantation is performed 3. Patients of ENKTCL show primary resistance to anthracycline-based regimens, mainly due to overexpression of multi-drug resistance (MDR) gene 4. Over the past decade, various regimens based on asparaginase have proved to be effective in ENKTCL, such as SMILE 5, GELOX 6, AspaMetDex 7, and DDGP 8. Notwithstanding the activity of asparaginase in ENKTCL, several patients demonstrate primary or secondary resistance to asparaginase, the prognosis of whom is extremely dismal. Although immunotherapy using anti-PD-1 antibodies have brought impressive benefits to relapsed/refractory patients of ENKTCL 9, asparaginase, as the cornerstone of ENKTCL treatment, is irreplaceable. Thus, it is of great importance to explore biomarkers predicting sensitivity to asparaginase and investigate mechanisms mediating asparaginase resistance.

Asparaginase kills ENKTCL by depriving tumor cells of L-asparagine, an important nutrient factor, which cannot be efficiently synthesized by ENKTCL cells due to their reduced L-asparagine synthetase (ASNS) level 10. Previous studies have revealed that ASNS level in patients of ENKTCL correlated with sensitivity to asparaginase 11. Meanwhile, asparaginase could also hydrolyze L-glutamine into L-glutamic acid and ammonia 12. Both L-asparagine and L-glutamine are important nutrient to ENKTCL cells, and upregulation of autophagy could be induced as a protective mechanism in response to nutrient withdrawal 13, 14. It has been demonstrated in acute myeloid leukemia 15, chronic myeloid leukemia 16, and ovarian cancer cells 17 that asparaginase can induce apoptosis as well as autophagy, and asparaginase-induced tumor cell death could be enhanced by inhibiting autophagic process. Thus, we hypothesized that autophagy might mediate resistance to asparaginase in ENKTCL.

Long non-coding RNAs (lncRNAs) are a class of non-coding RNAs with transcripts greater than 200 nucleotides, which have limited protein-coding potential. Increasing studies have revealed the critical role of lncRNAs in regulating expression or activity of genes involved in cancer via various mechanisms 18, 19. Brain cytoplasmic RNA 1 (BCYRN1), also known as BC200, has emerged as a valuable prognostic biomarker in several solid tumors, such as non-small cell lung cancer 20, breast cancer 21, liver cancer 22, and so on. Our previous study found a significant increase in expression of BCYRN1 in ENKTCL tumor tissue when compared to normal natural killer (NK) cells, especially in those with acquired resistance to asparaginase. Thus, in this present study, we evaluated the prognostic value of BCYRN1 in ENKTCL, and further investigated the detailed mechanisms of BCYRN1 in lymphomagenesis and mediating resistance to asparaginase.

## Materials and methods

### ENKTCL patients

40 patients newly diagnosed as ENKTCL were treated with P-GemOx 6 regimen (pegaspargase, gemcitabine, and oxaliplatin) in Beijing TongRen Hospital between January 2015 to December 2018. The diagnosis of ENKTCL was confirmed according to the 2008 WHO classification of Tumors of Haematopoietic and Lymphoid Tissues. Detailed clinical data and formalin fixed paraffin-embedded (FFPE) tissues were available in all 40 patients. In this cohort, three patients were defined as primary resistant to asparaginase since no responses were recorded after 2 cycles of treatment. NK cells in peripheral blood were collected from 10 healthy donors. The study procedure was approved by the Institutional Review Board (IRB) of Beijing TongRen Hospital. Informed consent for the collection of medical information was obtained from all patients at their first visit and the healthy donors.

### Cell lines and culture

The human ENKTCL cell lines SNK-6 and NK-92 cells were generously donated by professor Jin-xin Bei (Sun Yat-sen University Cancer Center). The SNK-6 cells were cultured in RPMI-1640 (Gibco, USA) medium supplemented with 10% fetal bovine serum (FBS; Gibco, USA) and 1000 U/mL interleukin (IL)-2 (Sigma-Aldrich, USA), simultaneously containing 2 mmol/L glutamine, 100 μg/mL streptomycin, and 100 U/mL penicillin in a humidified atmosphere of 5% CO_2_ incubator at 37 °C. The NK-92 cells were cultured in α-MEM (Life Technologies, Karlsruhe, Germany) with 20% FBS (Gibco, USA), 0.2 mM inositol, 0.1 mM 2-mercaptoethanol, 0.02 mM folic acid, 100 mg/mL penicillin, 2 mM L-glutamate, and 100 mg/mL streptomycin (Life Technologies) and supplemented with 150 U/mL IL-2 (Sigma-Aldrich, USA) in an incubator with 5% CO_2_ at 37 °C.

### Isolation of normal NK cells

Peripheral blood mononuclear cells (PBMCs), containing NK cells, were isolated from healthy donors' blood using the Ficoll density gradient centrifugation (Biocoll, Biochrom). Enrichment of NK cells were conducted via EasySep Human NK cell Enrichment Kit according to manufacturer's instructions (StemCell Technologies). The purification and characterization of NK cells were measured by flow cytometry, featured by CD16^+^, CD56^+^, CD69^+^, and CD3^-^. Freshly isolated NK cells were cultured in X-VIVO 10 medium (Lonza, Switzerland) supplemented with 5% heat-inactivated human plasma, 1% Pen/Strep and 10 ng/mL IL-15 (Peprotech) at a concentration of 2 × 10^6^ cells/mL (NK cell enrichment).

### Construction of SNK-6 cells with BCYRN1 overexpression or knockdown

Lentivirus vectors expressing BCYRN1 or shBCYRN1 were constructed as described previously 20. Briefly, the full-length BCYRN1 cDNA and step-loop for the shBCYRN1 was inserted into the LV5 vector (GenePharma Co. Ltd., China) at NotI and BamHI sites. The LV5-BCYRN1 vector, LV5-shBCYRN1 vector and LV5 control vector (LV5-NC) were, respectively, co-transfected with the packaging vectors pGag/Pol, pRev, and pVSV-G (GenePharma Co. Ltd., China) into HEK293T cells using Lipofectamine 2000 (Beyotime, Shanghai, China). The supernatant of the transfected HEK293T cells was collected 72 h after transfection. 2×10^4^ SNK-6 cells/well were infected for 24 h with the repackaged lentivirus with the polybrene (5 μg/mL). After replaced with fresh medium, the cells were cultured for 48 h, and the successfully infected cells were selected using puromycin (1.0 μg/mL) for 7-10 days prior to expansion.

### Transfection with p53 overexpression or knockdown plasmids

To construct the p53 overexpression plasmid, full-length human p53 cDNA was PCR-amplified using primers (Accession No: NM_000546.6). After digesting the PCR products with EcoRI and BamHI, the fragment was ligated into the EcoRI/BamHI sites of pcDNA3.1 to construct the pcDNA3.1-p53. All cDNA sequences were confirmed by Sanger sequencing. shRNA for p53 (shp53) was obtained from GenePharma (Shanghai, China). SNK-6 cells transfected with LV5-BCYRN1 and LV5-shBCYRN1 were transfected with pcDNA3.1-p53 and shp53 plasmid, respectively, by using Lipofectamine® 2000 (Invitrogen; Thermo Fisher Scientific, Inc.).

### RNA extraction and quantitative real-time PCR (qRT-PCR)

Total cellular RNA was extracted from 40 FFPE samples using the QIAGEN FFPE RNeasy kit (QIAGEN GmbH, Hilden, Germany). Total RNA was extracted from NK cells or cell lines by the TRIzol reagent method (Invitrogen, 15596018) following the manufacturer's protocol. PARIS^TM^ Kit (Invitrogen, USA) was used to extract nuclear and cytoplasmic RNA fractionation following the manufacturer's protocol. RNA quality was measured using the RNA 6000 Pico LabChip^®^ Kit on an Agilent 2100 Bioanalyser (Agilent technologies, Waldbronn, Germany). 1 μg RNA was reverse-transcribed by using the PrimeScript RT reagent Kit with gDNA Eraser (TaKaRa, DRR047A), then subjected to qRT-PCR reaction by use of the QuantiTect SYBR Green PCR kit (QIAgen, 204143) and LightCycler 2.0 system (Roche). Reactions were performed in a 20 μL volume with 10 μL 2×SYBR Green PCR Master Mix. The primer pair sequences for BCYRN1 and the housekeeping gene GAPDH are as follows: BCYRN1, forward 5′-GCCTGTAATCCCAGCTCTCA-3′ and reverse 5′-GGGTTGTTGCTTTGAGGGAA-3′; GAPDH, forward 5′-ACCACAGTCCATGCCATCAC-3′ and reverse 5′-TCCACCACCCTGTTGCTGTA-3′. Relative gene expression was normalized to GAPDH levels. The fold change in RNA level was calculated by the 2-ΔΔCt method with MxPro 4.00 (Stratagene). All experiments were done in triplicate.

### Protein extraction and western blot analysis

Total proteins were extracted using RIPA lysis buffer (Beyotime, Shanghai, China) supplied with protease inhibitor cocktail (MCE, USA) and phosphatase inhibitor cocktail (MCE, USA). Equal amounts of proteins were run on SDS-polyacrylamide gel, then transferred to polyvinylidene difluoride (PVDF) membrane (Millipore, IPFL00010), probed with different primary antibodies p21 (ab218311, 1:300), CyclinD1 (ab16663, 1:200), p53 (ab32389, 1:1000), LC3B (ab192890, 1:2000), p62 (ab109012, 1:10000), Beclin-1 (ab207612, 1:2000), Bax (ab32503, 1:2000), Bcl-2 (ab32124, 1:1000), PI3K (ab32089, 1:1000), pAKT (ab8805, 1:500), ATK (ab179463, 1:10000), pmTOR (ab109268, 1:2000), mTOR (ab2732, 1:2000) at 4 °C overnight. Then the membrane was incubated with horseradish peroxidase-linked secondary antibodies (Goat Anti-Rabbit IgG-HRP, ab205718, 1:10000) and detected with use of an enhanced chemiluminesence detection kit (Thermo, 32209). The relative quantity of proteins was analyzed by ImageJ software and normalized to loading controls. All experiments were done in triplicate.

### RNA-Fluorescence In-Situ Hybridization (RNA-FISH)

The expression and location of BCYRN1 in the asparaginase sensitive and resistant ENKTCL tumor tissues were determined by the fluorescence in-situ hybridization (FISH). Briefly, cells were grown on coverslips and fixed for 10 min in 4% formaldehyde at room temperature. Then the cells were rehydrated, digested and refixed in 4% paraformaldehyde. After that, the cells were prehybridized with hybridization solution (100 mg/mL dextran sulfate, 1 mg/mL E. coli tRNA, 2xSSC, 2 mM vanadyl ribonucleoside complex, 10% formamide, 0.2 mg/mL BSA, DEPC-H2O) and then incubated with a digoxigenin-labeled BCYRN1 probe at 42 °C for 20 h. Next day, the cells were washed with 2×SSC, 1×SSC and 0.5×SSC respectively, and then incubated with a mouse anti-digoxin antibody conjugated with alkaline phosphatase (Sigma-Aldrich, A1054). For visualizing the positive signal, the sections were mounted in antifade reagent (Vectashield Mounting medium with DAPI) and incubated in NBT/BCIP (Roche, 11681451001). Staining of treated samples was performed for an equal length of time. Images were captured using a DeltaVision RT microscope system (Applied Precision), and images were deconvolved and projected in two dimensions using SoftWoRx software.

### Immunohistochemistry staining

The slices of xenograft ENKTCL tissues were obtained. 3% hydrogen peroxide prepared with distilled water was used to seal the slices at room temperature for 10 min and then antigen repair was performed. 10% serum was prepared, and the slices were antigen sealed for 30 min. Then the serum was discarded, and anti-Ki-67 antibody (1:1000, ab15580, Abcam, USA) was added for incubation at 37 °C overnight. Secondary antibody (1:3000, 8885S, Cell Signaling Technology, MA) was added and incubated at room temperature for 1 h. After washing 4 times, add Vulcan Fast Red Chromogen Kit 2 staining for 15 min and terminate. Staining was carried out by adding DAB until all parts presented light yellow, and then terminated in distilled water.

### Detection of apoptosis in xenograft ENKTCL tissues by TUNEL assay

The formalin-fixed, paraffin-embedded 5μm sections of representative tumor samples (n=12) were studied by TUNEL (terminal deoxynucleotidyl transferase-mediated deoxyuridine triphosphate-biotin nick end labeling) staining with ApopTag Kit (InterGen, New York). The apoptotic rate was estimated by counting the positive brown-stained cells on 5 random high-power fields (original magnification ×400) from 3 tumor samples each of the 4 groups (n=12).

### Hoechst 33258 staining

Apoptosis was observed by using Hoechst apoptosis kit (Beyotime, China). SNK-6 cells (1×10^6^ cells/ml) were added onto the sterile cover glasses placed in the 6-well plates. 48 h after treated with specific drugs such as L-ASP, 3-MA, or Rapamycin, cells were fixed and washed twice with PBS. The Hoechst staining solution were prepared by diluting the Hoechst^®^ stock solution 1:2,000 in PBS. The medium was removed and the cells were stained with Hoechst 33258 staining solution according to the manufacturer's instructions. Remove the staining solution and wash the cells 3 times in PBS. Stained nuclei were observed under a fluorescence microscope.

### Cell proliferation assay

Cell proliferation assay was conducted by Cell Counting Kit-8 (CCK8) (Dojindo Lab, Japan). Briefly, 3000 cells/well were cultured in 96-well plate at 37 °C after each well was added with 10 μL CCK-8 solution. Then, the spectrophotometric absorbance was measured at 450 nm every day by Thermo Scientific Varioskan Flash machine for each sample. All the experiments were performed in triplicate and repeated three times, and the mean value was calculated.

### *In vitro* drug-sensitivity assay

For the drug-sensitivity assay in vitro, SNK-6 cells were seeded into 96-well plates with a density of 1×10^5^ cells/well. The culture medium containing different concentrations of L-ASP (0.01, 0.05, 0.25, 1.25, 6.25, 31.25, 156.25, 781.25 IU/mL) was added to each well. After 48 h, CCK-8 solution (10 μL per 100 µL of medium in each well) was added to each well and incubated for 2 h. The absorbance was measured by scanning with microplate reader (MRX; Dynex Technologies, West Sussex, United Kingdom) at 450 nm. Each group comprised six replicates, and the experiments were repeated 3 times. Then, the IC50 values for L-ASP were calculated.

### Clone formation experiment

In brief, cells (2×10^4^) transfected with LV5-NC, LV5-BCYRN1 and LV5-shBCYRN1 vector were respectively plated into three-well plates and cultured for two weeks. 10% formaldehyde was used to fix the colonies for 20 min and 0.1% crystal violet was used to stain for 10 min. The amount of colonies including ≥ 50 cells was counted through a microscope. All experiments were conducted three times.

### Flow cytometry analysis

Cells were harvested at 48 h after transfection. The FITC-Annexin V and propidium iodide (PI) double dyes were used to stain the cells by using the FITC Annexin V Apoptosis Detection Kit (BD Biosciences) according to the manufacturer's directions. After double staining, the cells were analyzed by flow cytometry (FACScan; BD Biosciences) equipped with CellQuest software (BD Biosciences). Cells were classified as viable, dead, early apoptotic, and apoptotic. To investigate cell cycle, the cells were stained with PI by using the CycleTEST Plus DNA Reagent Kit (BD Biosciences) following the protocol, and analysed by FACScan. The percentage of cells in G0/G1, S, and G2/M phase were counted. All experiments were done in triplicate.

### Immunofluorescence assay

In brief, cells were fixed with 4% paraformaldehyde for 15 min and blocked with 3% normal goat serum or rabbit serum for 20 min at room temperature. Then, the cells were incubated with Ad-GFP-LC3B primary antibody (1:100) at 4 °C overnight and then corresponding secondary antibody (1:200, Sangon Biotech, AB10051) at 37 °C for 1 h. Cells were washed 3 times with 0.1M phosphate-buffered saline (PBS: 2.7 mM KCl, 137 mM NaCl, 10 mM Na_2_HPO_4_, 2 mM KH_2_PO_4_) to eliminate the uncombined secondary antibody. The samples were evaluated by laser-scanning confocal microscopy (Leica, DMIRE2, Wetzlar, Germany).

### RNA-binding protein immunoprecipitation (RIP) assay

RIP assay was performed with a Magna RIP RNA-Binding Protein Immunoprecipitation Kit (Millipore) according to the manufactuer's protocal. Briefly, cells were harvested by adding RIP lysis buffer and incubated with protein beads and p53 antibody complex at 4 °C overnight. After washing off unbound materials, RNAs binding to p53 were eluted and quantified. We used qRT-PCR to examine certain RNAs coimmunoprecipitated with the p53 antibody.

### RNA pull-down assay

We used mpliScribe™ T7-Flash™ Biotin-RNA Transcription Kit (Epicentre, Madison, WI, USA) to transcribe and purify biotin labeled BCYRN1 probe in vitro. Then protein extracts (1 mg) was mixed with biotin-labelled RNAs (50 pmol) for 2 h, and incubated with streptavidin agarose beads (Invitrogen) at room temperature for 1 h. After stringent washing with the wash/binding buffer, the retrieved protein was analyzed by western blot.

### *In vivo* ubiquitination assay

Briefly, 293T cells were transfected with his-Ubiquitin and pcDNA3.0-BCYRN1 for 36 h and cells were harvested. Then, cells were denatured using denaturation buffer (6 M guanidine-HCl, 0.1 M Na_2_HPO_4_/NaH_2_PO_4_, 10 mM imidazole pH = 8) and cell lysates were incubated with pre-washed Ni-NTA Agarose beads (Qiagen) for 3.5 h to pull-down his-Ubiquitin and his-Ubiquitin-conjugated proteins. Beads were then washed with elution buffer (25 mM Tris-Cl, 20 mM Imidazole pH 6.8) and analyzed by Western blotting.

### Subcutaneous xenograft model

Sixty-four BALB/c nude mice aged 4-6 weeks (Nanjing Medical University Experimental Animal Center, Nanjing) were selected. These mice are adapted to housing conditions for 10 days, light/dark cycle for 12 h, relative humidity of 45-65%, temperature of 18-23 °C, diet and water can be used at will. These mice were randomly divided with 8 rats in each group. The SNK-6 cells infected with LV5-NC, LV5-BCYRN1 and LV5-shBCYRN1 were digested in routine, and washed with PBS for three times, and prepared into single cell suspension. Each group was divided into several equal fractions, each 100 µL (the total number of cells included was 1x10^5^). The cells transfected with LV5-NC, LV5-BCYRN1 and LV5-shBCYRN1 were inoculated subcutaneously into the buttocks of mice, respectively. In the treatment group, the mice were administered with intraperitoneal injection of asparaginase at a dose of 50 IU/day for 2 weeks once the tumor volume was measurable (about 3 days after inoculation). The tumor volume was measured on day 3, 7, 14, 21, and 28. All the mice were euthanized post experimentation, and the subcutaneous tumors were excised, weighed and the photos were taken. All animal experiments were performed according to the recommendations established by the US National Institutes of Health Guide for the Care and Use of Laboratory Animals. The study protocol was approved by the Animal Care and Ethics Committee of Capital Medical University.

### Statistical analysis

All data was presented as mean±SEM, mean values were derived from at least 3 independent experiments. The data was analyzed by SPSS 20 (SPSS Inc., Chicago, IL). Images were processed by use of Graphpad Prism 5 (GraphPad Software, Inc. La Jolla, CA, USA) and Adobe Photoshop CS3 (Adobe, San Jose, USA). For ENKTCL patients, progression-free survival (PFS) time was calculated from date of diagnosis to date of disease progression, death, or last follow up, whichever came first. Differences at p < 0.05 were considered statistically significant.

## Results

### Expression of BCYRN1 in ENKTCL and its prognostic value

**Figure 1:**
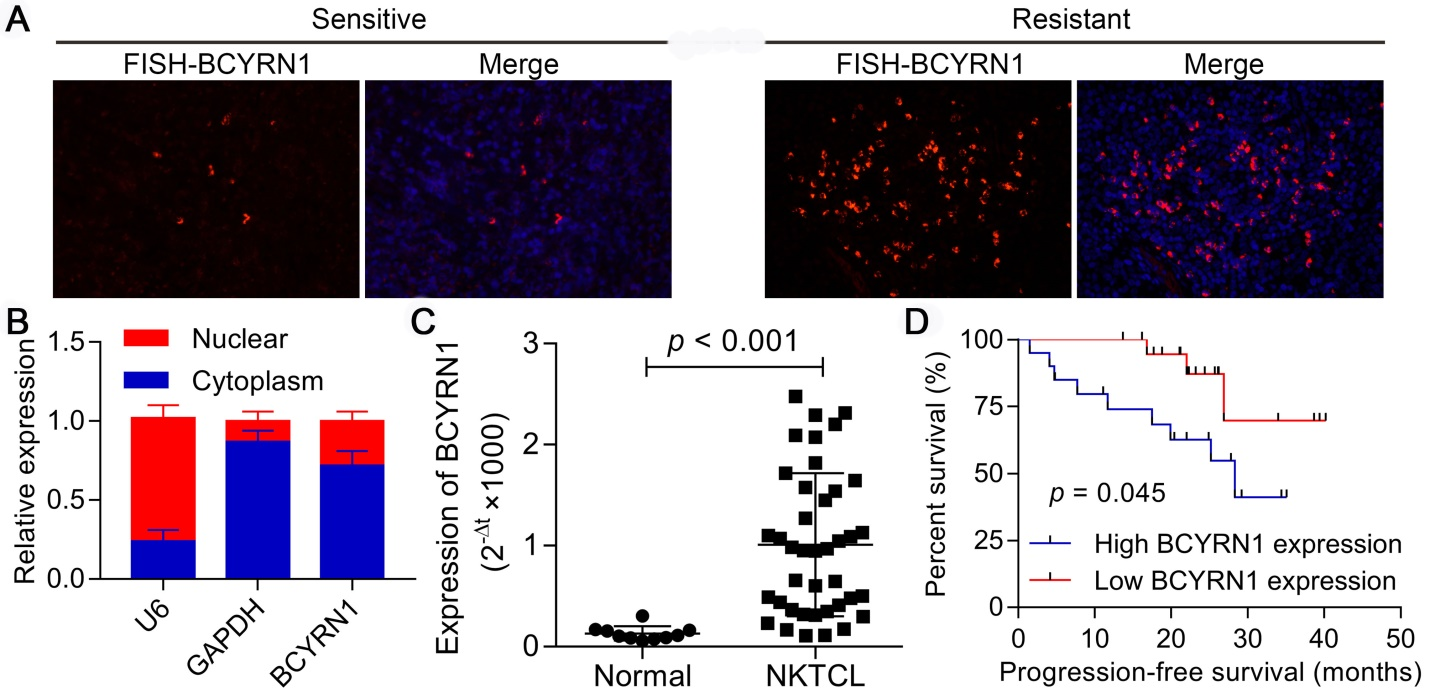
Expression of BCYRN1 in ENKTCL and its prognostic value. A. FISH assay of BCYRN1 in L-ASP sensitive and L-ASP resistant ENKTCL tissues. The L-ASP sensitive patient was a 46 years-old male patient with stage IIE ENKTCL, who got complete remission after two cycles of P-GemOx (pegaspargase, gemcitabine, and oxaliplatin). The L-ASP resistant patient was a 61 years-old male patient with stage IIE ENKTCL, who got progressive disease after 2 cycles of P-GemOx. Nuclei was stained blue with DAPI, and BCYRN1 was shown in red. B. Using U6 and GAPDH as internal references, BCYRN1 was found to be largely localized in the cytoplasm. C. The RNA level of BCYRN1 was detected in normal NK cells from 10 healthy donors and ENKTCL cells from 40 FFPE tissues. D. Kaplan-Meier analysis of progression-free survival according to BCYRN1 expression level.

Three of the forty patients with ENKTCL in our cohort were defined as primary resistant to asparaginase (they did not respond to asparaginase-based chemotherapy). We first investigated the expression status of BCYRN1 using FISH in these three resistant samples and three representative sensitive samples. As is demonstrated in Figure 1A, strikingly more cells in resistant patients showed intensive cytoplasmic positivity for BCYRN1 than in sensitive patients, which reflects the potential correlation between BCYRN1 expression and asparaginase resistance. Next, both nuclear and cytoplasmic RNA fractionation was extracted from SNK-6 cells. Using U6 and GAPDH as internal references, BCYRN1 was found to be largely localized in the cytoplasm (Figure 1B). Then, total cellular RNA was extracted from 40 FFEP ENKTCL samples and 10 healthy donors' NK cells. As shown in Figure 1C, expression of BCYRN1 was significantly higher in ENKTCL than normal NK cells (p < 0.001). Using the median expression level as the cutoff value, all 40 ENKTCL patients were classified into high BCYRN1 expression group and low BCYRN1 expression group (20 patients each group). Patients in high expression group had significantly inferior progression-free survival (PFS) than their counterparts (p = 0.045), indicating the potential prognostic role of BCYRN1 in ENKTCL (Figure 1D).

### Overexpression or knockdown of BCYRN1 affects ENKTCL cell proliferation, apoptosis, and cell cycle distribution in vitro

**Figure 2:**
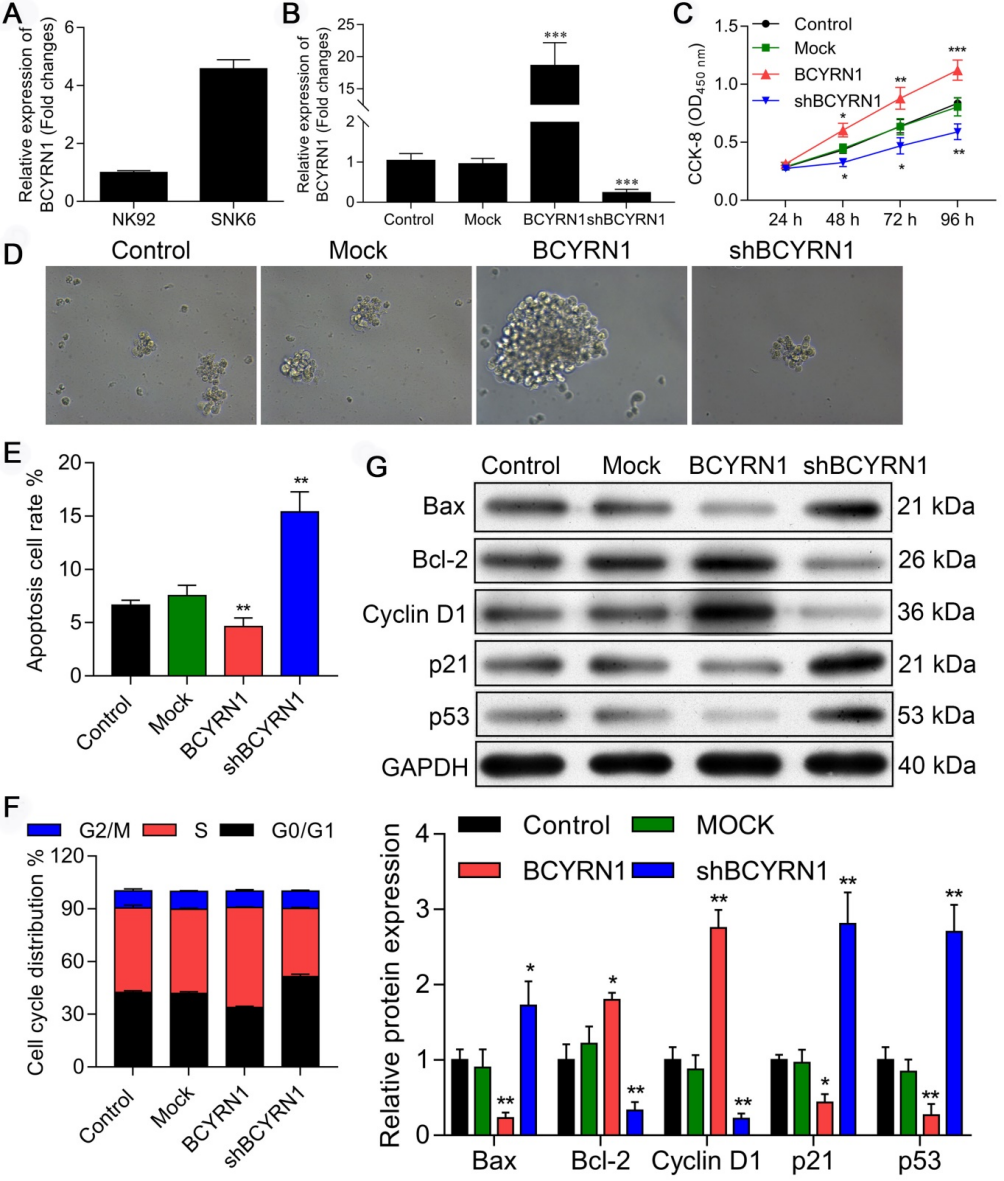
Impact of BCYRN1 on cell proliferation, apoptosis, and cell cycle distribution in SNK-6 cell line. A. The RNA level of BCYRN1 was detected in NK-92 and SNK-6 cell lines using RT-PCR. B. Lentivirus-mediated BCYRN1 overexpression and knockdown were performed in ENKTCL cell line SNK-6, and the RNA level of BCYRN1 was detected using RT-PCR. C and D. The cell viability and proliferation was measured by CCK8 (C) and clone formation experiment (D) in the SNK-6 cells with BCYRN1 overexpression or knockdown. E. The cell apoptosis was evaluated by annexin V/propidium iodide staining and quantified by the flow cytometry. F. The cell cycle distribution was measured and quantified by flow cytometry analysis. G. Apoptosis-related proteins Bax and Bcl-2, cell cycle-related proteins p21 and CyclinD1, and p53 were detected by Western blot. Control means normal SNK-6 cell lines; Mock indicates the SNK-6 cells transfected with empty virus vectors. BCYRN1 means SNK-6 cells transfected with BCYRN1-overexpressed lentivirus. shBCYRN1 means SNK-6 cells transfected with BCYRN1-knockdown lentivirus. *, p < 0.05; **, p < 0.01; ***, p < 0.001.

The RNA level of BCYRN1 was examined in both SNK-6 cell line and NK-92 cell line using qRT-PCR. As shown in Figure 2A, BCYRN1 was significantly overexpressed in SNK-6 cells than in NK-92 cells. Next, to determine whether BCYRN1 affects ENKTCL cell growth in vitro, SNK-6 cells were stably transfected by lentivirus of BCYRN1-overexpression (labeled as BCYRN1) or BCYRN1-knockdown (labeled as shBCYRN1). The overexpression and knockdown efficiency in the transfected SNK-6 cells were confirmed by qRT-PCR (Figure 2B). The cell viability and proliferation of SNK-6 cells in different groups were determined by CCK-8 (Figure 2C) and clone formation experiments (Figure 2D), which demonstrated that BCYRN1 overexpression could promote SNK-6 cell proliferation. By using annexin V/propidium iodide staining and flow cytometry analysis, the apoptosis rate was calculated. As is shown in Figure 2E and Figure S1A, knockdown of BCYRN1 could significantly induce apoptosis in SNK-6 cells, which was validated by Hoechst 33258 staining (Figure S1B). Then cell cycle distribution was measured and quantified by flow cytometry (Figure 2F and Figure S1C). Overexpression of BCYRN1 resulted in more cells entering into S phase, while knockdown of BCYRN1 blocked cell cycle to G0/G1 phase. As revealed in Figure 2G, several apoptosis and cell cycle related proteins were detected using western blot. It can be seen that BCYRN1 overexpression significantly increased the expression of apoptotic inhibitory protein Bcl-2 and cell cycle factor CyclinD1, whereas p53 and the pro-apoptotic protein Bax were significantly decreased. Thus, the above experimental results indicated that overexpression of BCYRN1 could enhance the proliferation of SNK-6 cells by reducing cell apoptosis and promoting the cells to enter S phase. On the contrary, knockdown of BCYRN1 could induce apoptosis and block cells to the G0/G1 phase, and eventually partially inhibit the proliferation of SNK-6 cells.

### Impact of BCYRN1 overexpression on drug resistance of SNK-6 cells to asparaginase

**Figure 3:**
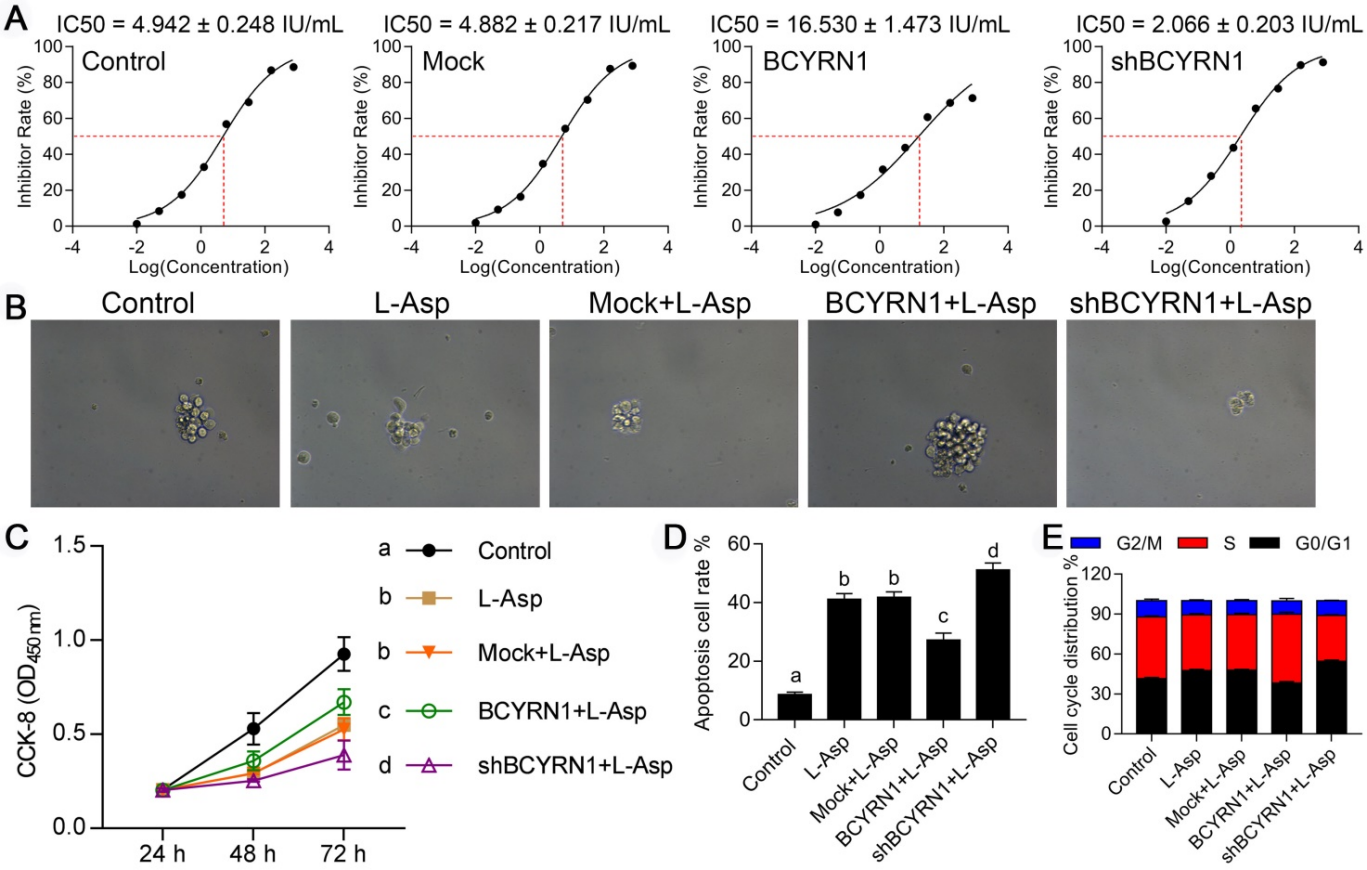
Impact of BCYRN1 on drug resistance of SNK-6 cells to asparaginase. A. The IC50 values for asparaginase in different groups of SNK-6 cells were calculated using CCK8 method. B and C. The cell viability and proliferation was measured by clone formation experiment (B) and CCK8 (C) in different groups of SNK-6 cells. D. The cell apoptosis was evaluated by annexin V/propidium iodide staining and quantified by the flow cytometry. E. The cell cycle distribution was measured and quantified by flow cytometry analysis. Control means normal SNK-6 cell lines; Mock indicates the SNK-6 cells transfected with empty virus vectors. BCYRN1 means SNK-6 cells transfected with BCYRN1-overexpressed lentivirus. shBCYRN1 means SNK-6 cells transfected with BCYRN1-knockdown lentivirus. *, p < 0.05; **, p < 0.01; ***, p < 0.001. In C and D, different letters represent significant differences between groups.

SNK-6 cell line was sensitive to asparaginase with IC50 value of 4.942 IU/mL. The IC50 value of BCYRN1-overexpressed SNK-6 cells was significantly increased to 16.530 IU/mL (Figure 3A), indicating resistance to asparaginase was conferred by BCYRN1 overexpression. By CCK-8 and clone formation experiments (Figure 3B-C), it can be seen that BCYRN1 overexpression could resist the inhibitory effect of asparaginase on proliferation and clone formation. As is shown in Figure S2A-B and Figure 3D, asparaginase induced significant apoptosis of SNK-6 cells, which could be partially counteracted by BCYRN1 overexpression and facilitated by BCYRN1 knockdown. Cell cycle distribution analysis demonstrated that asparaginase blocked cell cycles to G0/G1, while BCYRN1 overexpression could push more cells reenter S phase (Figure 3E, Figure S3C). Thus, the above findings indicated that overexpression of BCYRN1 could promote drug resistance to asparaginase, while knockdown of BCYRN1 might enhance the sensitivity to asparaginase in SNK-6 cells.

### Overexpression of BCYRN1 induced autophagy by suppressing PI3K/AKT/mTOR and p53/mTOR pathways

**Figure 4:**
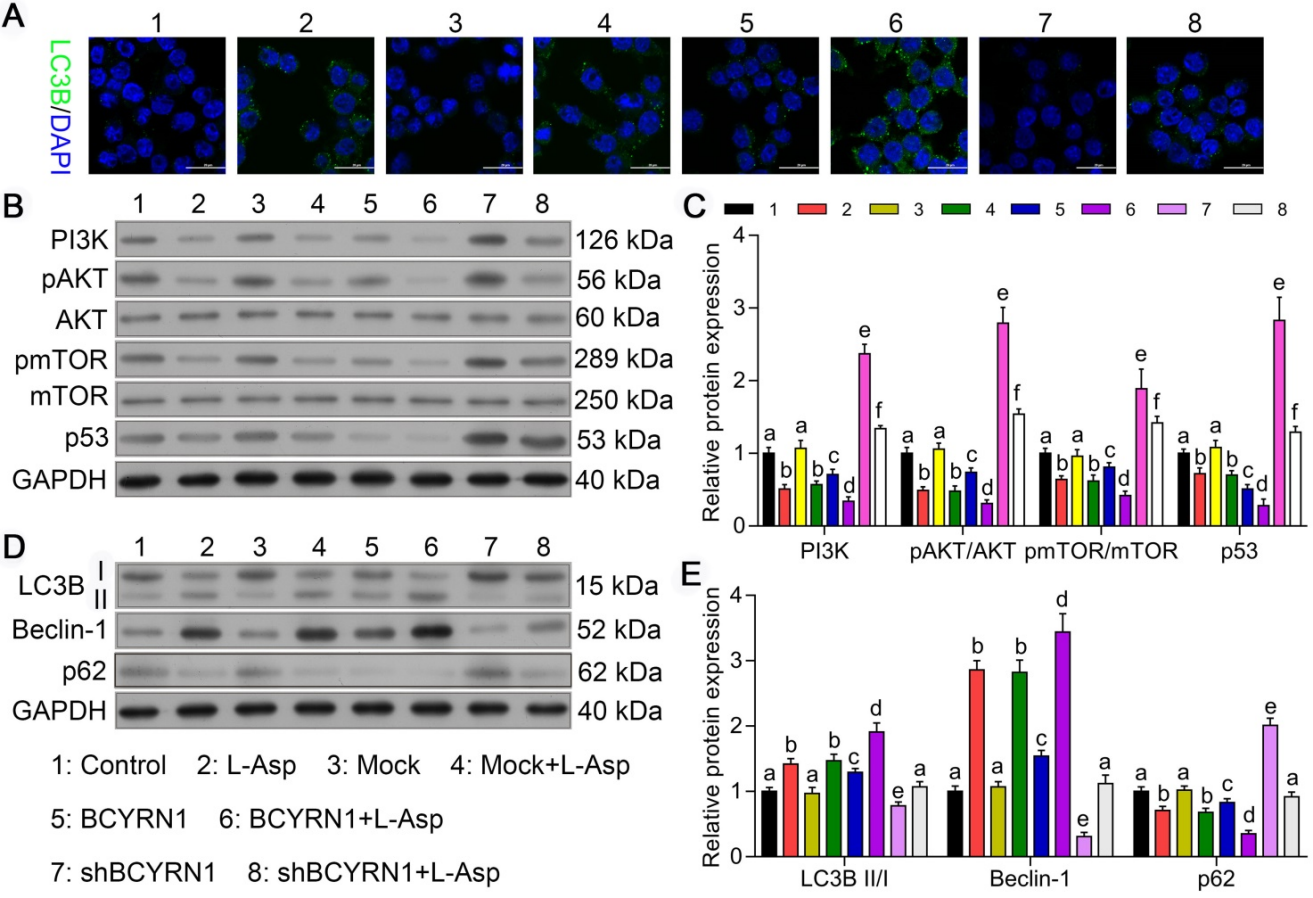
BCYRN1 induced autophagy by suppressing PI3K/AKT/mTOR and p53/mTOR pathways. A. Cell autophagy was analyzed by immunofluorescence assay with adenovirus expressing GFP-LC3B fusion protein (Ad-GFP-LC3B) in different groups of SNK-6 cells. Nuclei was stained blue with DAPI, and LC3B was shown in green. B, and C. Western blot analysis was performed to assess the impact of BCYRN1 on the proteins expression of PI3K/AKT/mTOR and p53/mTOR pathways. D, and E. The expression of autophagy-related proteins was detected by Western blot analysis. Control means normal SNK-6 cell lines; Mock indicates the SNK-6 cells transfected with empty virus vectors. BCYRN1 means SNK-6 cells transfected with BCYRN1-overexpressed lentivirus. shBCYRN1 means SNK-6 cells transfected with BCYRN1-knockdown lentivirus. L-ASP means SNK-6 cells treated with asparaginase. In C and E, different letters represent significant differences between groups.

Autophagy, a lysosome-mediated intracellular degradation system, which can be induced by chemotherapeutics, could protect cancer cells during exogenous drugs stress conditions, thereby contributing to chemoresistance. Therefore, we hypothesized that the resistance of SNK6 cells to asparaginase enhanced by BCYRN1 may have increased autophagy. As we mentioned above, asparaginase can induce concurrent apoptosis and autophagy in other malignancies 23. Thus, we used adenovirus expressing GFP-LC3B fusion protein (Ad-GFP-LC3B) to transfect SNK-6 cells, and autophagy status was detected by immunofluorescent assay. As shown in Figure 4A, compared to control or Mock cells, SNK-6 cells treated with asparaginase showed obvious autophagy, which was significantly enhanced by overexpression of BCYRN1 and diminished by BCYRN1 knockdown. Using western blot analysis, we found that both PI3K/AKT/mTOR and p53/mTOR pathways were inhibited by asparaginase treatment and BCYRN1 overexpression (Figure 4B-C), thus leading to enhancement of autophagy, which was validated by detection of Beclin-1 and conversion of LC3-I to LC3-II (Figure 4D-E). Beclin-1 plays a central role in autophagy, and autophagosome formation is definitely related with conversion of LC3 from LC3-I to the LC3-II form. Thus, significantly increased expression of Beclin-1 and LC3-II in BCYRN1-overexpressed or asparaginase-treated SNK cells indicated the enhancement of autophagy. From the above findings, we inferred that BCYRN1 could lead to asparaginase resistance by inducing autophagy, which might be mediated by inhibiting PI3K/AKT/mTOR and p53/mTOR pathways.

### BCYRN1 facilitated the ubiquitination of p53 by direct interaction

**Figure 5:**
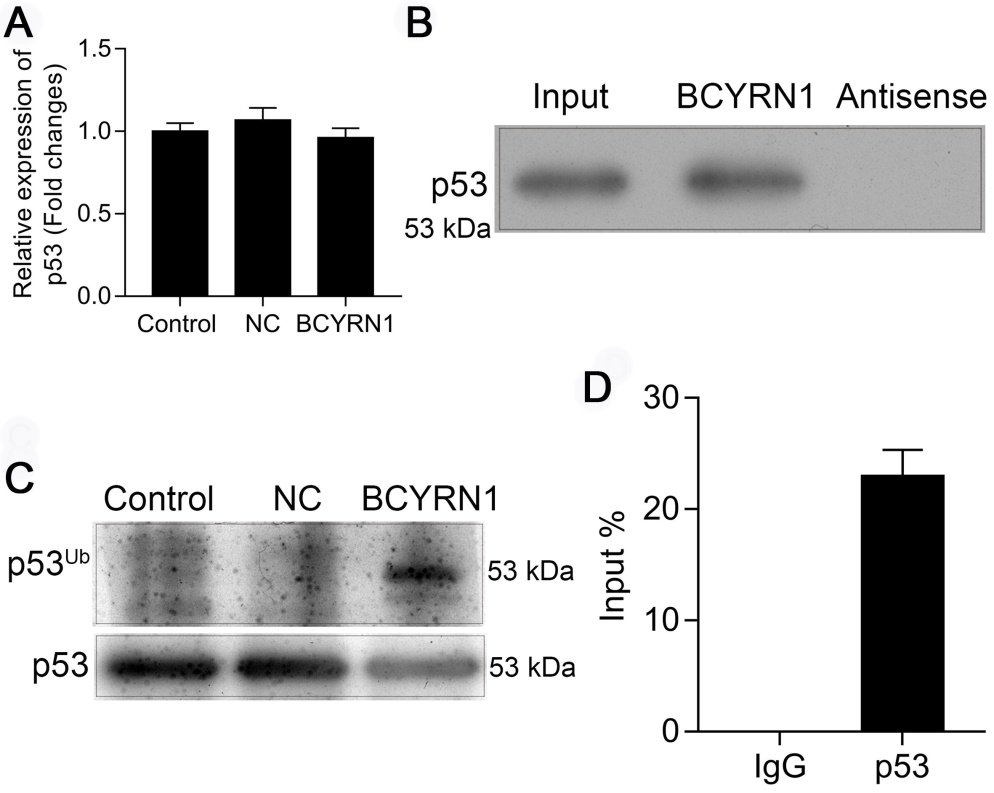
BCYRN1 facilitated the ubiquitination of p53. A. The mRNA level of p53 was measured using qRT-PCR in BCYRN1-overexpressed SNK-6 cells, which demonstrated that overexpression of BCYRN1 did not affect the mRNA expression of p53. B. RNA pull-down assay followed by western blot analysis for candidate protein p53 in SNK-6 cells. C. 293T cells were co-transfected with his-ubiquitin plasmid (His-Ub) and BCYRN1 plasmid for 36 h before in vivo ubiquitination assay to access the p53 ubiquitination. D. RIP assay for the binding of candidate protein p53 with BCYRN1. RIP was performed using anti-p53 antibody, followed by qRT-PCR assay for BCYRN1 expression in SNK-6 cells.

It is well recognized that post-translational modifications play critical roles in controlling the dynamic balance of p53 in normal cells, and ubiquitination of p53 is the first defined pathway affecting p53 protein levels and turnover. To determine how BCYRN1 affected the expression level and function of p53, we firstly measured the mRNA level of p53 in BCYRN1-overexpressed SNK-6 cells using qRT-PCR. As is shown in Figure 5A, BCYRN1 did not affect the RNA level of p53, indicating the possibility of post-translational modifications. Then we performed RNA-binding protein immunoprecipitation (RIP) assay and RNA pull-down assay to see whether BCYRN1 interacts with p53 or not. As is shown in Figure 5B-C, BCYRN1 could interact with p53 in vitro. By in vivo ubiquitination assay, we demonstrated that BCYRN1 could facilitate the ubiquitination of p53, resulting in its degradation (Figure 5D).

### Overexpression of p53 or inhibition of autophagy eliminated BCYRN1-mediated resistance to asparaginase

**Figure 6:**
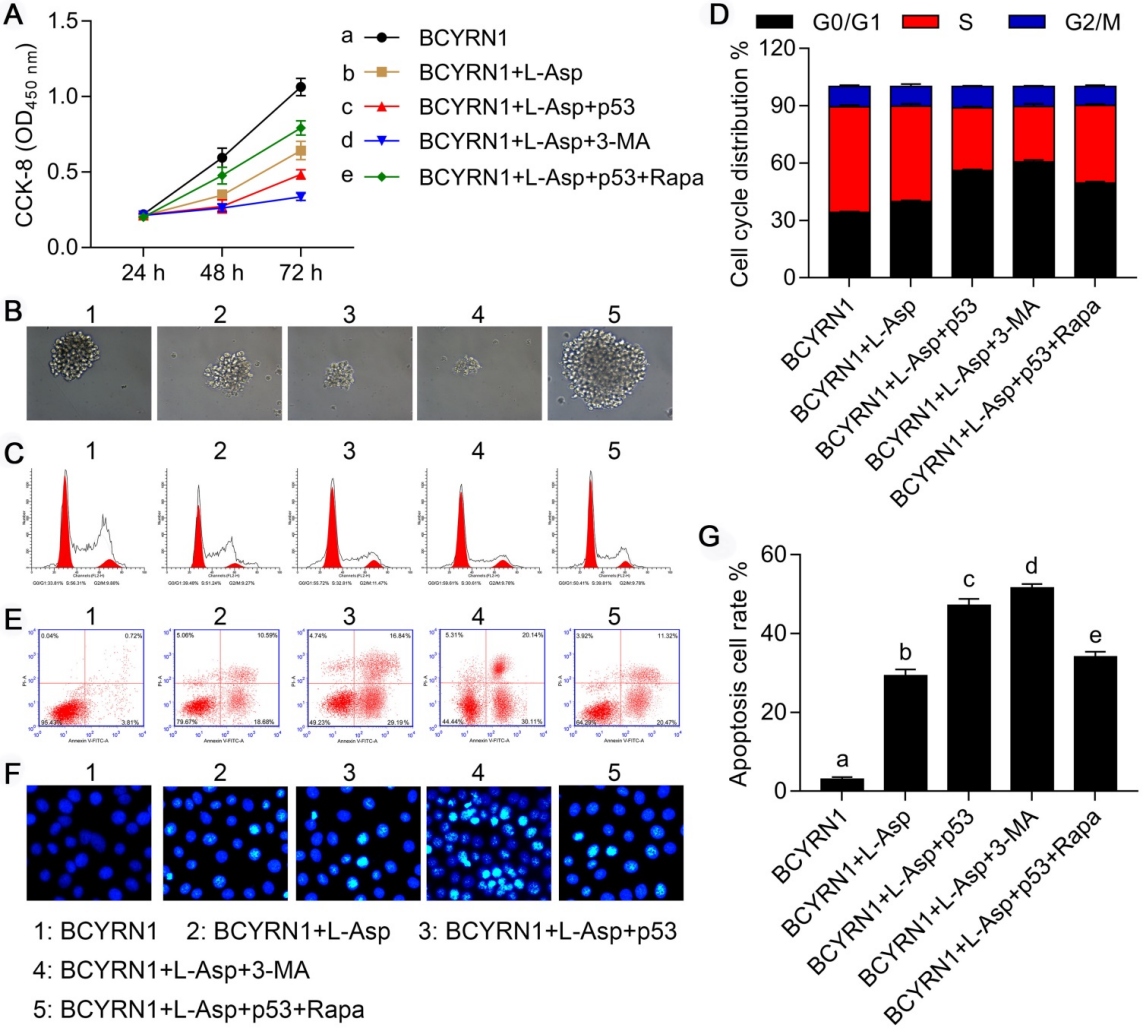
Overexpression of p53 or inhibition of autophagy eliminated BCYRN1-mediated resistance to asparaginase. A and B. The cell viability and proliferation was measured by CCK8 (A) and clone formation experiment (B) in different groups of SNK-6 cells. C and D. The cell cycle distribution was measured and quantified by flow cytometry analysis. E, F, and G. The cell apoptosis was evaluated by annexin V/propidium iodide staining and quantified by the flow cytometry (E, G) and Hoechst 33258 staining (F) (The nuclei of apoptotic cells are bright blue and lobulated or fragmented). BCYRN1 means SNK-6 cells transfected with BCYRN1-overexpressed lentivirus. L-ASP means SNK-6 cells treated with asparaginase. p53 means BCYRN1-overexpressed SNK-6 cells transfected with pcDNA3.1-p53 plasmid. 3-MA means SNK-6 cells treated with 3-MA. Rapa means SNK-6 cells treated with Rapa. In A, D and G, different letters represent significant differences between groups.

**Figure 7:**
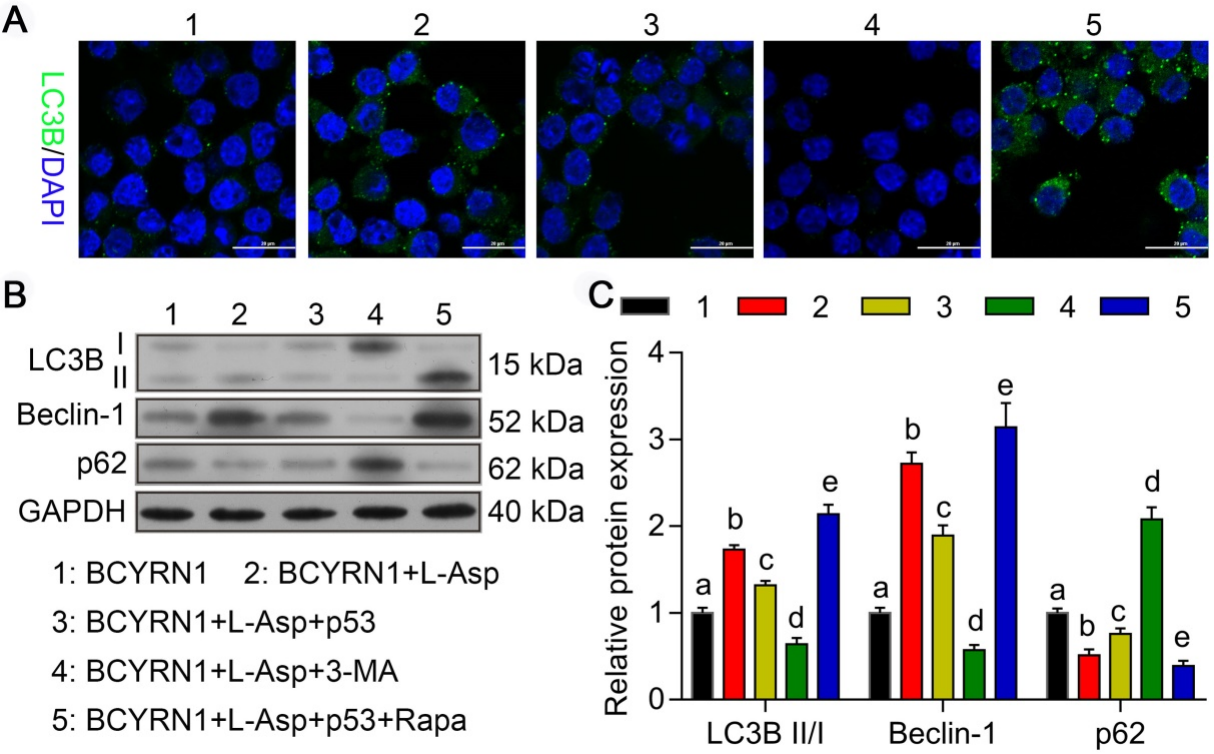
Overexpression of p53 or autophagy inhibitors inhibit BCYRN1-induced autophagy. A. Cell autophagy was analyzed by immunofluorescence assay with adenovirus expressing GFP-LC3B fusion protein (Ad-GFP-LC3B) in different groups of SNK-6 cells. Nuclei was stained blue with DAPI, and LC3B was shown in green. B, and C. The expression of autophagy-related proteins was detected by Western blot analysis. BCYRN1 means SNK-6 cells transfected with BCYRN1-overexpressed lentivirus. L-ASP means SNK-6 cells treated with asparaginase. p53 means BCYRN1-overexpressed SNK-6 cells transfected with pcDNA3.1-p53 plasmid. 3-MA means SNK-6 cells treated with 3-MA. Rapa means SNK-6 cells treated with Rapa. In C, different letters represent significant differences between groups.

To evaluate the role of autophagy in the process of resistance to asparaginase, we constructed p53-overexpressed (labeled as p53) and -knockdown (labeled as sh-p53) plasmids to transiently transfect BCYRN1-SNK-6 cells and shBCYRN1-SNK-6 cells, respectively. 3-Methyladenine (3-MA), a selective PI3K inhibitor, could inhibit autophagosome formation via the inhibition of class III PI3K, especially under nutrient-deprived conditions^24^, and rapamycin (Rapa) can induce autophagy by directly inhibiting mTOR activation. As is shown in Figure 6 and Figure 7, overexpression of p53 inhibited cell proliferation and clone formation, induced cell apoptosis and cell cycle blockade to G0/G1 phase, probably due to inhibition of autophagy, which was confirmed by decreased expression of LC3-II and Beclin-1. Similarly, by inhibiting autophagy using 3-MA, the sensitivity to asparaginase was significantly enhanced in BCYRN1-SNK-6 cells. On the contrary, Rapa promoted autophagy by directly inhibiting mTOR activation, and eventually diminished the inhibitory role of p53 overexpression on SNK-6 cell proliferation. Chloroquine (CQ), a classic autophagy inhibitor, could block autophagosome fusion and degradation. As is shown in Figure S3, addition of 10μmol/L CQ could sensitize BCYRN1-overexpressed SNK-6 cells to ASP (IC50 value = 7.757 IU/mL). Thus, we confirmed our hypothesis that BCYRN1 induced resistance of SNK-6 cells to asparaginase by promoting autophagy, and this scenario could be reversed by autophagy inhibition.

### BCYRN1 promoted ENKTCL growth and resistance to asparaginase in vivo

**Figure 8:**
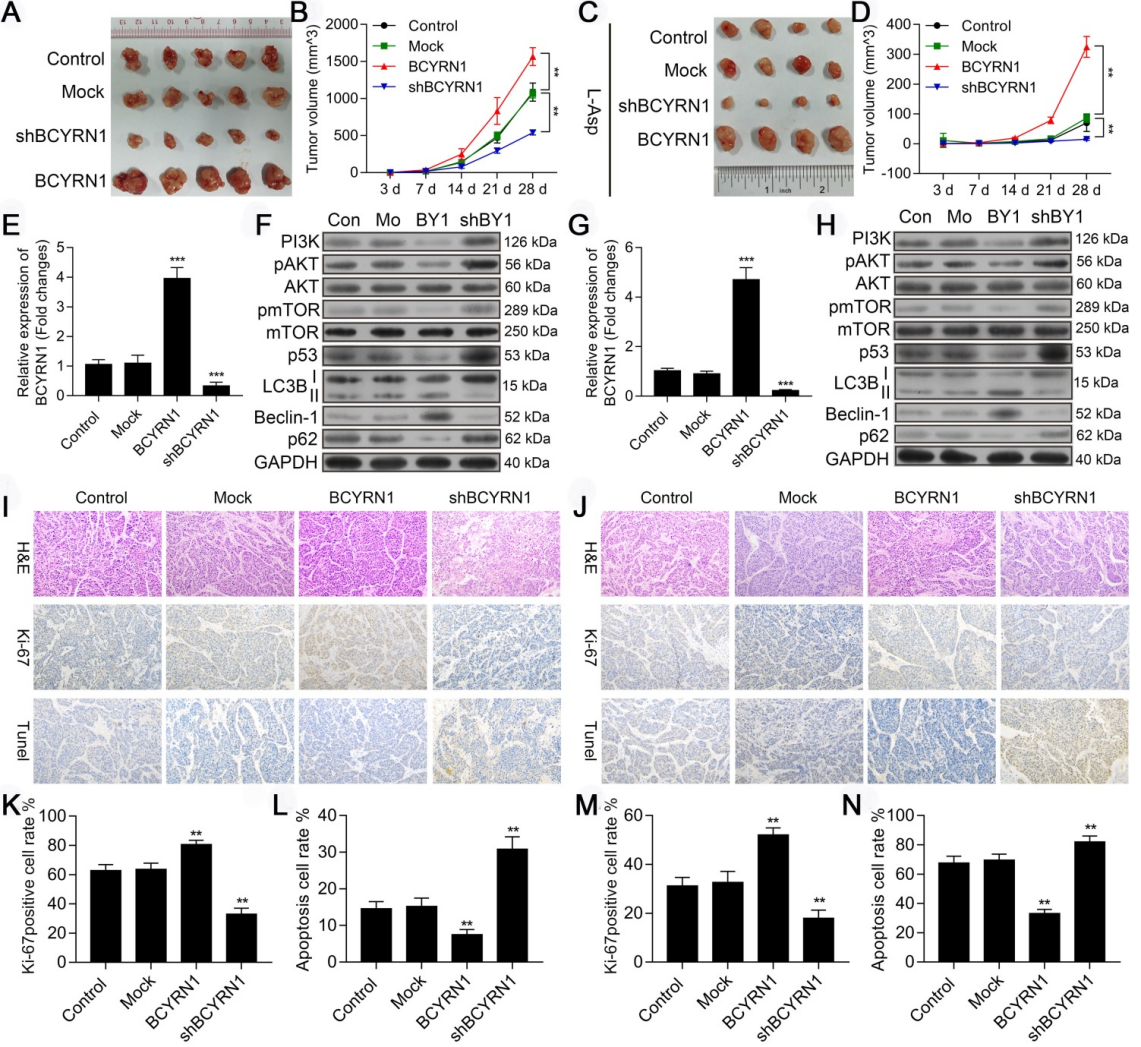
Impact of BCYRN1 on ENKTCL tumor growth and autophagy in vivo. A and C. The image of tumors in xenograft tumor models treated without (A, n = 5 in each group) or with asparaginase (C, n = 4 in each group). Normal SNK-6 cells and SNK-6 cells transfected with LV5-NC, LV5-BCYRN1 and LV5-shBCYRN1 were inoculated subcutaneously into the buttocks of mice to establish the xenograft tumor model. B and D. Tumor volume was measured on day 3, 7, 14, 21, and 28 in xenograft tumor models treated without (B) or with asparaginase (D). E and G. The RNA level of BCYRN1 in xenograft tumor tissues treated without (E) or with asparaginase (G) using RT-PCR. F and H. Expression of proteins involved in the PI3K/AKT/mTOR and p53/mTOR pathways and autophagy-related proteins were detected by western blot analysis in xenograft tumor tissues treated without (F) or with asparaginase (H). I and J. Immunohistochemistry staining of Ki-67 and TUNEL assay in xenograft tumor tissues treated without (I) or with asparaginase (J). Staining of the nucleus in brown is defined as Ki-67 positive. The apoptotic cells are brown-stained. K and M. Ki-67 proliferation index in different xenograft tumor tissues treated without (K) or with asparaginase (M), which is measured by counting 500 cells and reported as percent positive. L and N. Apoptosis rate was calculated in different xenograft tumor tissues treated without (L) or with asparaginase (N). Control (Con) means normal SNK-6 cell lines; Mock (Mo) indicates the SNK-6 cells transfected with empty virus vectors; BCYRN1 (BY1) means SNK-6 cells transfected with BCYRN1-overexpressed lentivirus; shBCYRN1 (shBY1) means SNK-6 cells transfected with BCYRN1-knockdown lentivirus. *, p < 0.05; **, p < 0.01; ***, p < 0.001.

To confirm the in vitro activity of BCYRN1, we constructed ENKTCL xenograft models using SNK-6 cells stably transfected with BCYRN1 or shBCYRN1. In the treatment group, intraperitoneal injection of asparaginase was given when subcutaneous tumors were measurable (about 3 days after cell implantation) at a dose of 50 IU/d for the subsequent 14 days. As is shown in Figure 8A (control) and C (treatment group), ENKTCL xenograft models were sensitive to asparaginase treatment, and the gross tumor volum was significantly smaller than models without asparaginase treatment. In BCYRN1-overexpressed xenograft models, tumor growth rate was significantly higher than BCYRN1-knockdown models (Figure 8B and 8D). Using qRT-PCR, we confirmed the efficiency of BCYRN1 overexpression and knockdown in xenograft models (Figure 8E and 8G). Similar as findings in vitro, BCYRN1 overexpression enhanced expression of Beclin-1 and LC3-II by decreasing expression of PI3K/AKT/mTOR and p53/mTOR pathway-related proteins (Figure 8F and 8H). Ki-67, as a nuclear protein that is expressed in all stages of cell proliferation (G1, S, and M phase) but not in G0 phase, is often used as a marker of cell proliferation degree. Next, xenograft tumor sample sections were prepared for HE staining, ki-67 staining and TUNEL assay. As is shown in Figure 8I-N, BCYRN1-overexpressed tumor samples demonstrated high percentage of positivity and intensity of ki-67, indicating enhanced tumor proliferation. TUNEL assay revealed that the apoptotic rate was significantly increased after knockdown of BCYRN1 and treatment with asparaginase. Thus, this in vivo study verified that BCYRN1 could lead to resistance of ENKTCL to asparaginase by enhancing autophagy of tumor cells. By interfering expression of BCYRN1, the sensitivity of asparaginase could be restored, and potential synergistic effects further enhance the killing effect of asparaginase on ENKTCL.

## Discussion

**Figure 9:**
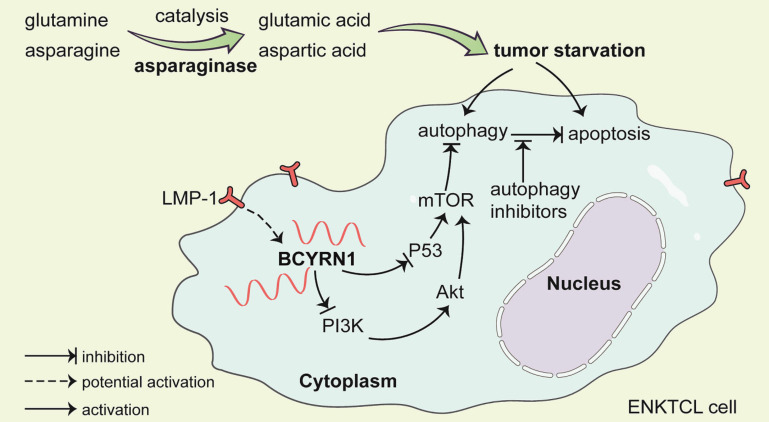
A schematic model of BCYRN1 in the resistance of ENKTCL to asparaginase. In this model, asparaginase causes ENKTCL cell starvation by depriving of asparagine and glutamine, which results in both apoptosis and autophagy, and the latter process acts as cell protective to exogenous stress. BCYRN1 that might be induced by LMP-1, promotes autophagy by suppressing both PI3K/AKT/mTOR and p53/mTOR pathways, and eventually enhances the resistance of ENKTCL to asparaginase, which can be reversed by drug-induced autophagy inhibition.

Since asparaginase was first introduced in the treatment of ENKTCL in 2003 25, it has become the cornerstone and shifted the treatment paradigm. Combination of asparaginase-based chemotherapy and radiotherapy cured about 80% of patients with early stage disease 2. The remaining 20% of early stage patients and the majority of advanced disease patients fare dismal due to primary or acquired resistance to asparaginase-based therapy. Thus, it is critical to identify those who are prone to be resistant to asparaginase at diagnosis and novel immunotherapy may be applied upfront to improve their prognosis. Moreover, mechanisms mediating resistance to asparaginase should be clarified in order to maximizing the tumor killing effect of asparaginase and discovering novel therapeutic targets. In this study, we reported for the first time that BCYRN1, a lncRNA, was both a valuable prognostic factor and predictive biomarker for sensitivity to asparaginase. Furthermore, BCYRN1 was elucidated to promote drug resistance of ENKTCL to asparaginase by enhancing autophagy, which could be reversed by drug induced autophagy inhibition (Figure 9).

BCYRN1, also known as BC200, was first discovered in 1987 and initial studies mainly focused on its role in brain development and neurodegenerative diseases 26. Until only recent years, increasing studies have uncovered the role of BCYRN1 in tumorigenesis and metastasis in various solid tumors 27. Compared to normal NK cells, expression of BCYRN1 was significantly increased in ENKTCL, and the detailed mechanism of its overexpression was not investigated in our present study. It has been demonstrated that MYC drives BCYRN1 expression in non-small cell lung cancer 20, but it remains to be seen whether MYC plays similar roles in ENKTCL, although MYC is dysregulated in various types of cancer, including ENKTCL 28. Both SNK-6 and NK-92 cells are originated from ENKTCL patients, but significant differences exist concerning the expression level of latent membrane protein-1 (LMP-1), which is strongly expressed in SNK-6 cells and weakly expressed in NK-92 cells. LMP-1, as the oncogenic gene of EBV, can be detected in nearly all patients of ENKTCL 29. Our study demonstrated that the RNA level of BCYRN1 was significantly higher in SNK-6 cells than in NK-92 cells, indicating a potential causal relationship between BCYRN1 expression and LMP-1, which warrants further investigation.

In consistent with the findings in solid tumors 20, 21, 30, BCYRN1 was found to be a cancer-promoting gene in ENKTCL, which accelerated tumor proliferation, reduced tumor cell apoptosis, and forced cell cycles into S phase. Patients with high expression level of BCYRN1 had significantly shorter PFS than their counterparts, indicating the potential oncogenic nature of BCYRN1 in ENKTCL. Using digoxigenin-labeled BCYRN1, we can visually see that asparaginase-resistant samples had much more and stronger expression of BCYRN1, which might partially explain the adverse prognostic role of BCYRN1 in ENKTCL.

Asparaginase is highly effective in the treatment of acute lymphocytic leukemia (ALL) and ENKTCL by depriving tumor cells of L-asparagine, which cannot be sufficiently synthesized by those tumors due to lack of enough ASNS 10. Thus, expression level of ASNS may indicate sensitivity to asparaginase, which has been proved in several previous studies 11. However, controversial results exist, and Hermanova et al did not conclude the correlation between low level of ASNS and asparaginase sensitivity in ALL patients 31. Thus, the predictive role of ASNS needs to be validated in larger cohort of patients with ENKTCL. As expected, treatment with asparaginase resulted in concurrent apoptosis and autophagy in SNK-6 cells. Autophagy is a double-edged sword in tumor, as it can promote or suppress tumor development depending on specific tumor types, microenvironment, and external stress 32, 33. Starvation caused by L-asparagine and L-glutamine deprivation can activate the process of autophagy as a tumor cell protective strategy, which in turn resist the killing effect of asparaginase 16. In this study, we found that BCYRN1 overexpression can enhance the process of autophagy in SNK-6 cells through inhibiting both PI3K/AKT/mTOR and p53/mTOR pathways. mTOR, a classic upstream molecule of autophagy pathway, activation of which can inhibit multiple autophagy related proteins via phosphorylation 34. Through inhibition of phosphorylated AKT and downstream activation of mTOR, overexpressed BCYRN1 significantly increased the expression of LC3-II and Beclin-1, both of which can reflect the process of autophagy. P53, a critical tumor suppressive gene, has dual functions in regulating autophagy, depending on its subcellular location 35, 36. In nucleus, p53 exerts pro-autophagic function by inhibiting mTOR phosphorylation through activation of AMP-activated protein kinase. However, in the cytoplasm, p53 can inhibit autophagy in a transcription-independent manner by inhibiting glycolysis and production of reactive oxygen species (ROS) 37. In our study, BCYRN1 was mainly located in cytoplasm, where it binds p53 and results in degradation of p53 through ubiquitination. Thus, overexpression of BCYRN1 mainly down-regulated the cytoplasmic level of p53, and eventually resulted in enhancement of autophagy. However, whether BCYRN1 could affect the function of nuclear p53 or not needs to be researched further. Moreover, the detailed binding site of BCYRN1 and p53, as well as other possible post-translational modifications of p53, should be investigated in future.

As the cornerstone drug for the treatment of ENKTCL, asparaginase-based chemotherapies have significantly improved the prognosis of ENKTCL patients 2, 6, 38-40. For those who failed asparaginase, immunotherapy using anti-PD-1 antibodies has emerged as a promising approach 9. As we can conclude from our study, drug-induced inhibition of autophagy may resensitize those patients to asparaginase. Chloroquine (CQ) and the related hydroxychloroquine (HCQ) are the two drugs mostly used in clinical trials to target autophagy 41. Most of these clinical trials have confirmed the safety profiles and provided clinical benefits in specific types of cancer 42. Meanwhile, inhibition of autophagy may increase the level of PD-L1 in gastric cancer cells through the p62/SQSTM1-NF-κB pathway, which in turn may enhance the effect of anti-PD-1 antibodies 43. Thus, autophagy inhibition plus asparaginase and/or anti-PD-1 antibodies seem to be an attractive combination for future investigation in relapsed/refractory ENKTCL patients.

However, there are some limitations in our study. Firstly, Xiong et al 44 has recently proposed a molecular subtyping system based on analyzing genomic and transcriptomic features of ENKTCL, among which the MB subtype (defined by MGB mutation and loss of heterozygosity in BRDT, and featured by increased MYC expression) has the worst prognosis due to resistance to commonly used chemotherapy drugs. Whether MYC could upregulated BCYRN1 in ENKTCL needs to be investigated further. Secondly, overexpression of BCYRN1 was demonstrated to promote ENKTCL cell proliferation in our research, and whether BCYRN1 could mediate resistance to other cytostatic drugs besides asparaginase (such as gemcitabine and cisplatin) should be explored further. Lastly, various mechanisms besides autophagy dysregulation may exist involving the drug resistance process, which warrants further investigation in ENKTCL.

## Conclusions

Our study found for the first time that BCYRN1 was a valuable prognostic factor and predictive biomarker for efficacy of asparaginase in patients with ENKTCL. BCYRN1 could promote drug resistance to asparaginase by inducing autophagy through PI3K/AKT/mTOR and p53/mTOR pathways in ENKTCL cell lines, and the sensitivity to asparaginase could be reversed by BCYRN1 knockdown or drug-induced inhibition of autophagy. Our findings highlight the feasibility of combining autophagy inhibition and asparaginase in the treatment of relapsed/refractory ENKTCL patients.

## Supplementary Material

Supplementary figures.

## Abbreviations

ENKTCL: Extranodal NK/T-cell lymphoma, nasal type
EBV: Epstein-barr virus
OS: overall survival
MDR: multi-drug resistance
ASNS: L-asparagine synthetase
LncRNAs: Long non-coding RNAs
BCYRN1: Brain cytoplasmic RNA 1
NK: natural killer
FFPE: formalin fixed paraffin-embedded
PBMCs: Peripheral blood mononuclear cells
qRT-PCR: quantitative real-time PCR
FISH: Fluorescence In-Situ Hybridization
TUNEL: terminal deoxynucleotidyl transferase-mediated deoxyuridine triphosphate-biotin nick end labeling
RIP: RNA-binding protein immunoprecipitation
PFS: progression-free survival
Ad-GFP-LC3B: adenovirus expressing GFP-LC3B fusion protein
3-MA: 3-Methyladenine
Rapa: rapamycin
LMP-1: latent membrane protein-1
CQ: Chloroquine
HCQ: hydroxychloroquine
