# Supplementary material for: LncRNA BCYRN1-induced autophagy enhances asparaginase resistance in extranodal NK/T-cell lymphoma
Source: Theranostics. 2021 Jan 1;11(2):925–40. doi: 10.7150/thno.46655 (PMC7738865; doi:10.7150/thno.46655)
Supplement: Supplementary file 1 — Supplementary figures. [file thnov11p0925s1.pdf]

Figure S1. Impact of BCYRN1 on cell apoptosis and cell cycle distribution in SNK-6 cell line.

A. The cell apoptosis was evaluated by annexin V/propidium iodide staining and quantified by the flow cytometry. B. The cell apoptosis was observed by Hoechst 33258 staining (The nuclei of apoptotic cells are bright blue and lobulated or fragmented). C. The cell cycle distribution was measured and quantified by flow cytometry analysis. Control means normal SNK-6 cell lines; Mock indicates the SNK-6 cells transfected with empty virus vectors. BCYRN1 means SNK-6 cells transfected with BCYRN1-overexpressed lentivirus. shBCYRN1 means SNK-6 cells transfected with BCYRN1-knockdown lentivirus.

Figure S2. Impact of BCYRN1 on drug resistance of SNK-6 cells to asparaginase.

A. The cell apoptosis was evaluated by flow cytometry analysis using annexin V/propidium iodide staining. B. The cell apoptosis was demonstrated by Hoechst 33258 staining (The nuclei of apoptotic cells are bright blue and lobulated or fragmented). C. The cell cycle distribution was measured by flow cytometry analysis. Control means normal SNK-6 cell lines; Mock indicates the SNK-6 cells transfected with empty virus vectors. BCYRN1 means SNK-6 cells transfected with BCYRN1-overexpressed lentivirus. shBCYRN1 means SNK-6 cells transfected with BCYRN1-knockdown lentivirus. L-ASP means SNK-6 cells treated with asparaginase.

Figure S3. Overexpression of p53 or autophagy inhibitors inhibit BCYRN1-induced autophagy.

A, B, and C. The cell viability and proliferation was measured by CCK8 (A, B) and clone formation experiment (C) in different groups of SNK-6 cells. D. The cell cycle distribution was measured and quantified by flow cytometry analysis. E, and F. The cell apoptosis was quantified by the flow cytometry (E) and Hoechst 33258 staining (F) (The nuclei of apoptotic cells are bright blue and lobulated or fragmented). H. Cell autophagy was analyzed by immunofluorescence assay with adenovirus expressing GFP-LC3B fusion protein (Ad-GFP-LC3B) in different groups of SNK-6 cells. Nuclei was stained blue with DAPI, and LC3B was shown in green. BCYRN1 means SNK-6 cells transfected with BCYRN1-overexpressed lentivirus. L-ASP means SNK-6 cells treated with asparaginase. p53 means BCYRN1-overexpressed SNK-6 cells transfected with pcDNA3.1-p53 plasmid. CQ means SNK-6 cells treated with Chloroquine (CQ). Rapa means SNK-6 cells treated with Rapa. In B and F, different letters represent significant differences between groups.

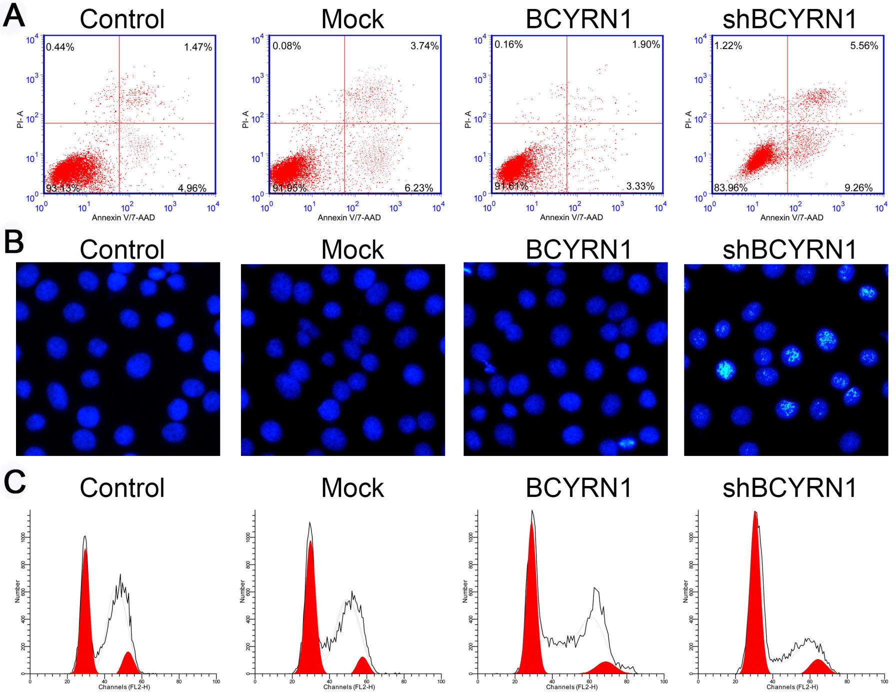

A
Control
Mock
BCYRN1
shBCYRN1
0.44%
1.47%
93.13%
4.96%
0.08%
3.74%
91.95%
6.23%
0.16%
1.90%
91.61%
3.33%
1.22%
5.56%
83.96%
9.26%
PI-A
Annexin V/7-AAD
B
Control
Mock
BCYRN1
shBCYRN1
C
Control
Mock
BCYRN1
shBCYRN1
Number
Channels (FL2-H)

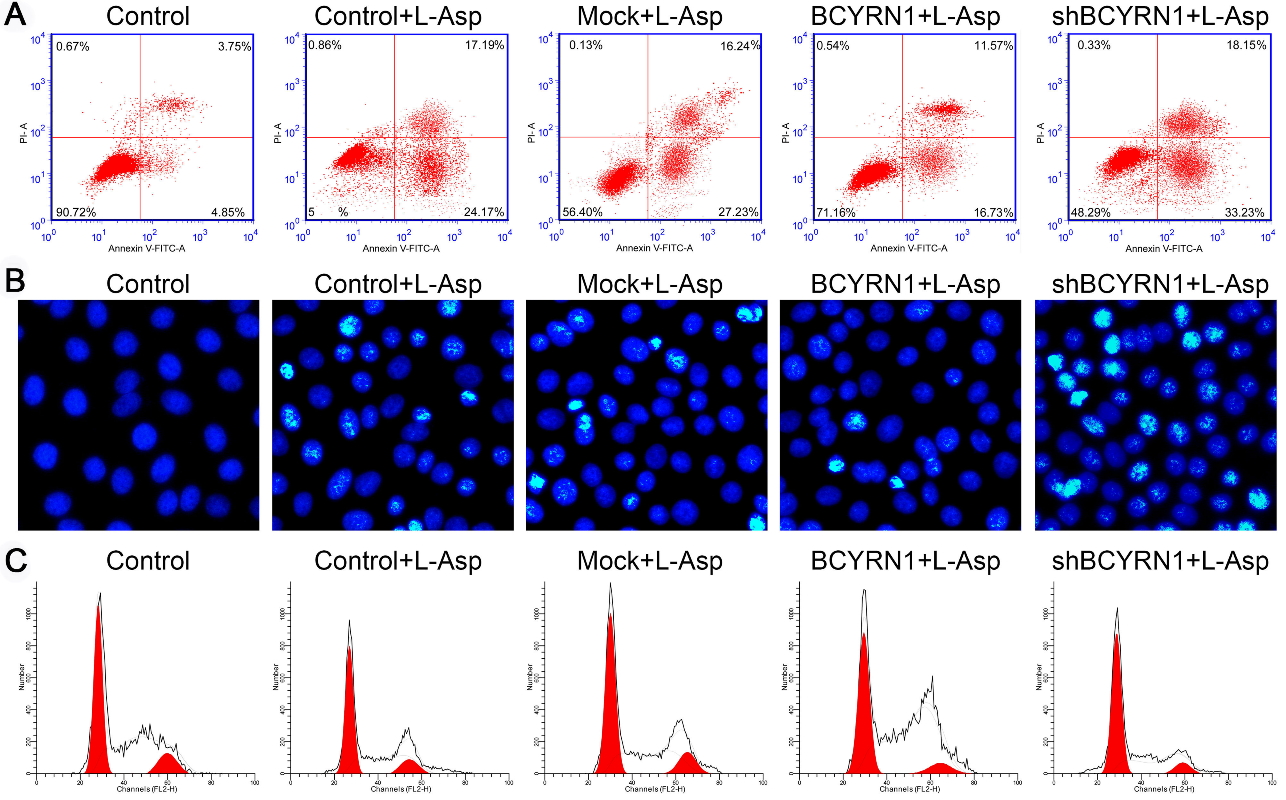
A
Control
Control+L-Asp
Mock+L-Asp
BCYRN1+L-Asp
shBCYRN1+L-Asp
0.67%
3.75%
90.72%
4.85%
0.86%
17.19%
24.17%
0.13%
16.24%
56.40%
27.23%
0.54%
11.57%
71.16%
16.73%
0.33%
18.15%
48.29%
33.23%
PI-A
Annexin V-FITC-A
B
Control
Control+L-Asp
Mock+L-Asp
BCYRN1+L-Asp
shBCYRN1+L-Asp
C
Control
Control+L-Asp
Mock+L-Asp
BCYRN1+L-Asp
shBCYRN1+L-Asp
Number
Channels (FL2-H)

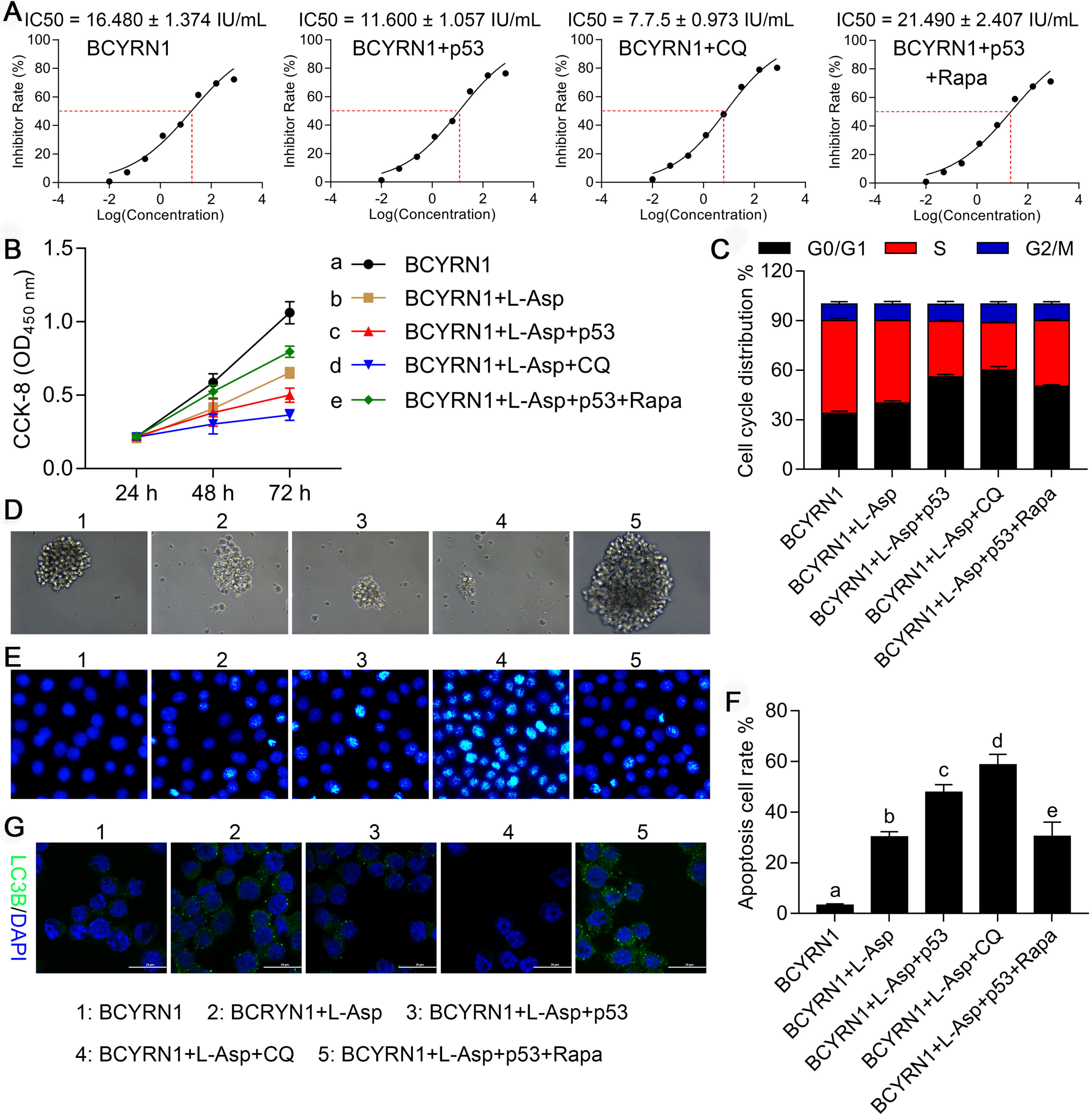

A
IC50 = 16.480 ± 1.374 IU/mL
BCYRN1
IC50 = 11.600 ± 1.057 IU/mL
BCYRN1+p53
IC50 = 7.7.5 ± 0.973 IU/mL
BCYRN1+CQ
IC50 = 21.490 ± 2.407 IU/mL
BCYRN1+p53 +Rapa
Inhibitor Rate (%)
Log(Concentration)
B
CCK-8 (OD450 nm)
24 h
48 h
72 h
a BCYRN1
b BCYRN1+L-Asp
c BCYRN1+L-Asp+p53
d BCYRN1+L-Asp+CQ
e BCYRN1+L-Asp+p53+Rapa
C
G0/G1
S
G2/M
Cell cycle distribution %
BCYRN1
BCYRN1+L-Asp
BCYRN1+L-Asp+p53
BCYRN1+L-Asp+CQ
BCYRN1+L-Asp+p53+Rapa
D
E
F
Apoptosis cell rate %
a
b
c
d
e
G
LC3B/DAPI
1: BCYRN1
2: BCRYN1+L-Asp
3: BCYRN1+L-Asp+p53
4: BCYRN1+L-Asp+CQ
5: BCYRN1+L-Asp+p53+Rapa
